# Reprogramming of Androgen Receptor Activity in Castration-resistant Prostate Cancer is Shaped by Truncated Variants

**DOI:** 10.1016/j.euf.2025.03.017

**Published:** 2025-04-11

**Authors:** Mitchell G. Lawrence, Shivakumar Keerthikumar, Scott L. Townley, Ashlee K. Clark, Georgia B. Cuffe, Geraldine Laven-Law, Adrienne R. Hanson, Raj K. Shrestha, Todd P. Knutson, Michelle G. Richards, Linda Teng, Nicholas Choo, Megan Crumbaker, Anthony M. Joshua, Eva Corey, Peter S. Nelson, Scott M. Dehm, Gail P. Risbridger, Wayne D. Tilley, Theresa E. Hickey, Renea A. Taylor, Luke A. Selth

**Affiliations:** a Department of Anatomy and Developmental Biology, Biomedicine Discovery Institute, Monash University, Clayton, Australia;; b Peter MacCallum Cancer Centre, Melbourne, Australia;; c Sir Peter MacCallum Department of Oncology, University of Melbourne, Melbourne, Australia;; d Cabrini Institute, Cabrini Health, Malvern, Australia;; e Melbourne Urological Research Alliance, Monash University, Clayton, Australia;; f Flinders University College of Medicine and Public Health, Flinders Health and Medical Research Institute, Bedford Park, Australia;; g Dame Roma Mitchell Cancer Research Laboratories, Adelaide Medical School, University of Adelaide, Adelaide, Australia;; h Minnesota Supercomputing Institute, University of Minnesota, Minneapolis, MN, USA;; i Garvan Institute of Medical Research, Sydney, Australia;; j St. Vincent’s Clinical School, UNSW Australia, Sydney, Australia;; k The Kinghorn Cancer Centre, St. Vincent’s Hospital, Sydney, Australia;; l Department of Urology, University of Washington, Seattle, WA, USA;; m Divisions of Human Biology and Clinical Research, Fred Hutchinson Cancer Center, Seattle, WA, USA;; n Masonic Cancer Center and Departments of Laboratory Medicine and Pathology and of Urology, University of Minnesota, Minneapolis, MN, USA;; o Department of Physiology, Biomedicine Discovery Institute Cancer Program, Monash University, Clayton, Australia;; p Freemasons Centre for Male Health and Wellbeing, Flinders University, Bedford Park, Australia;; q Faculty of Health and Medical Sciences, University of Adelaide, Adelaide, Australia

**Keywords:** Prostate cancer, Androgen receptor, Bipolar androgen therapy, Androgen deprivation therapy, Patient-derived xenograft, Chromatin immunoprecipitation, Cistrome

## Abstract

**Background and objective:**

Under the selective pressure of treatment, prostate cancer cells express constitutively active androgen receptor (AR) variants. Whether AR variants mediate therapy resistance remains contested, because they are often coexpressed with abundant full-length AR. Therefore, we sought to determine how truncated variants shape AR chromatin occupancy and responses to treatments in both the presence and absence of full-length AR.

**Methods:**

We used a cohort of patient-derived xenografts of metastatic prostate cancer with diverse *AR* alterations. Chromatin immunoprecipitation and RNA sequencing were used to compare the landscape of AR binding and transcriptomic features. We assessed responses to castration by castrating host mice and evaluated responses to bipolar androgen therapy by administering testosterone cypionate.

**Key findings and limitations:**

By profiling the AR cistrome, we identified a distinct group of tumours defined by ARv567es expression, a variant arising due to structural rearrangements of the *AR* gene. ARv567es-positive tumours also had a distinct epigenomic profile and altered transcriptional features, including loss of canonical AR-regulated gene signatures and elevated expression of AR-repressed genes. ARv567es-positive tumours were resistant to castration and bipolar androgen therapy. In tumours that coexpress full-length AR, this involves dampened transcriptional responses and disruption of the autoregulatory loop that modulates *AR* levels. Study limitations include the need for additional models of AR-driven prostate cancer.

**Conclusions and clinical implications:**

The emergence of ARv567es via gene rearrangements causes transcriptional reprogramming and therapy resistance. This highlights ARv567es as a potential as a marker to guide treatment decisions.

## 1. Introduction

In most cases of castration-resistant prostate cancer (CRPC), the androgen receptor (AR) remains a driver of tumour growth [1]. Numerous mechanisms of resistance sustain AR signalling in CRPC, including elevated expression of AR variants (AR-Vs) [2]. AR-Vs lack all or part of the ligand-binding domain but retain the transcriptionally active N-terminal domain and DNA-binding domain. Hence, they can regulate transcription independent of androgens [2]. AR-Vs are generated via two mechanisms: rapid, reversible induction of *AR-V* transcripts via alternative splicing of *AR* pre-mRNA [3], and/or structural rearrangements within the *AR* gene that promote high-level expression of AR-Vs [4]. The most common AR-V arising from alternative splicing is AR-V7, whereas the archetypal AR-V arising from *AR* genomic structural rearrangements (AR-GSRs) is ARv567es [5].

The clinical relevance of AR-Vs as drivers of therapy resistance remains controversial [6,7]. One reason for this uncertainty is that AR-Vs are often expressed alongside high levels of full-length AR (AR-FL), making it difficult to dissect their relative contributions [8]. AR-V mRNA levels are generally orders of magnitude lower than AR-FL transcript levels [8,9]. The coexpression of AR-Vs with AR-FL has also hindered attempts to determine how closely the AR-V-regulated transcriptional programme resembles that regulated by AR-FL [10].

We addressed these unresolved questions using patient-derived xenografts (PDXs) with AR-GSRs that result in high levels of ARv567es expression. The results demonstrate that ARv567es has distinct DNA binding and transcriptional activity and is uncoupled from androgen-mediated autoregulation, and that ARv567es-positive tumours are resistant to castration and bipolar androgen therapy (BAT).

## 2. Patients and methods

### 2.1. Patient-derived xenografts

PDXs were previously established in accordance with human and animal ethics approvals [11–13] (Supplementary materials and methods). To model androgen deprivation therapy (ADT), PDXs were grafted in testosterone-supplemented mice. Mice were then castrated and their testosterone pellets were removed. The PDXs were subsequently serially regrafted into castrated mice. To model BAT, PDXs were grafted in castrated mice, which were then treated with vehicle (sesame oil) or 1 mg of testosterone cypionate via intramuscular injection every 2 wks [14]. Treatment was terminated if tumours reached 1000 mm^3^.

### 2.2. Chromatin immunoprecipitation sequencing

PDXs were processed for chromatin immunoprecipitation sequencing (ChIP-seq) as previously described [15]. We used a pool of two antibodies for AR (Abcam 74272, 3 μg; Millipore PG-21, 2 μg), and Abcam 4729 (2 μg) for H3K27ac. Libraries were prepared using an Ultra Low Library Preparation Kit (Qiagen) and sequenced on a NextSeq OH platform (Illumina; single-end 75 bp). The Supplementary material describes the data processing in detail.

### 2.3. Immunohistochemistry

Immunohistochemistry (IHC) was performed using a BOND-MAX automated system (Leica Biosystems). Antibodies and staining conditions are listed in Supplementary Table 1. Staining was assessed using H scores [16].

### 2.4. Quantitative reverse transcriptase-polymerase chain reaction and RNA sequencing

Total RNA was isolated using RNeasy kits (Qiagen). Quantitative reverse transcriptase-polymerase chain reaction (qRT-PCR) was performed using a Power SYBR Green Master Mix or TaqMan RT-PCR Assay (ThermoFisher Scientific). Relative expression was calculated using the ΔΔCt method and the geometric mean for *RPLPO*, *TMEM199*, and *ZNF207* reference genes [12]. The primers used are listed in Supplementary Table 2 [17]. RNA sequencing was performed with the NEB NextSeq HO stranded protocol (75-bp paired-end reads; Illumina). Genes with differential expression between ARv567es-positive and AR-v567es-negative PDXs were filtered using false discovery rate <0.01 or log fold change ≥2 or ≤ −2. Single-sample gene-set enrichment analyses were calculated using the *GSVA* package in R. The Supplementary material provides full details.

### 2.5. AR-targeted DNA sequencing

DNA was analysed using a custom *AR*-targeted DNA sequencing assay [4,18]. AR-GSRs were identified via consensus between two structural variant callers (LUMPY and Delly). Data were visualised with Integrative Genomics Viewer (https://igv.org/).

## 3. Results

### 3.1. The AR cistrome becomes heterogeneous with cancer progression

**Fig. 1 –:**
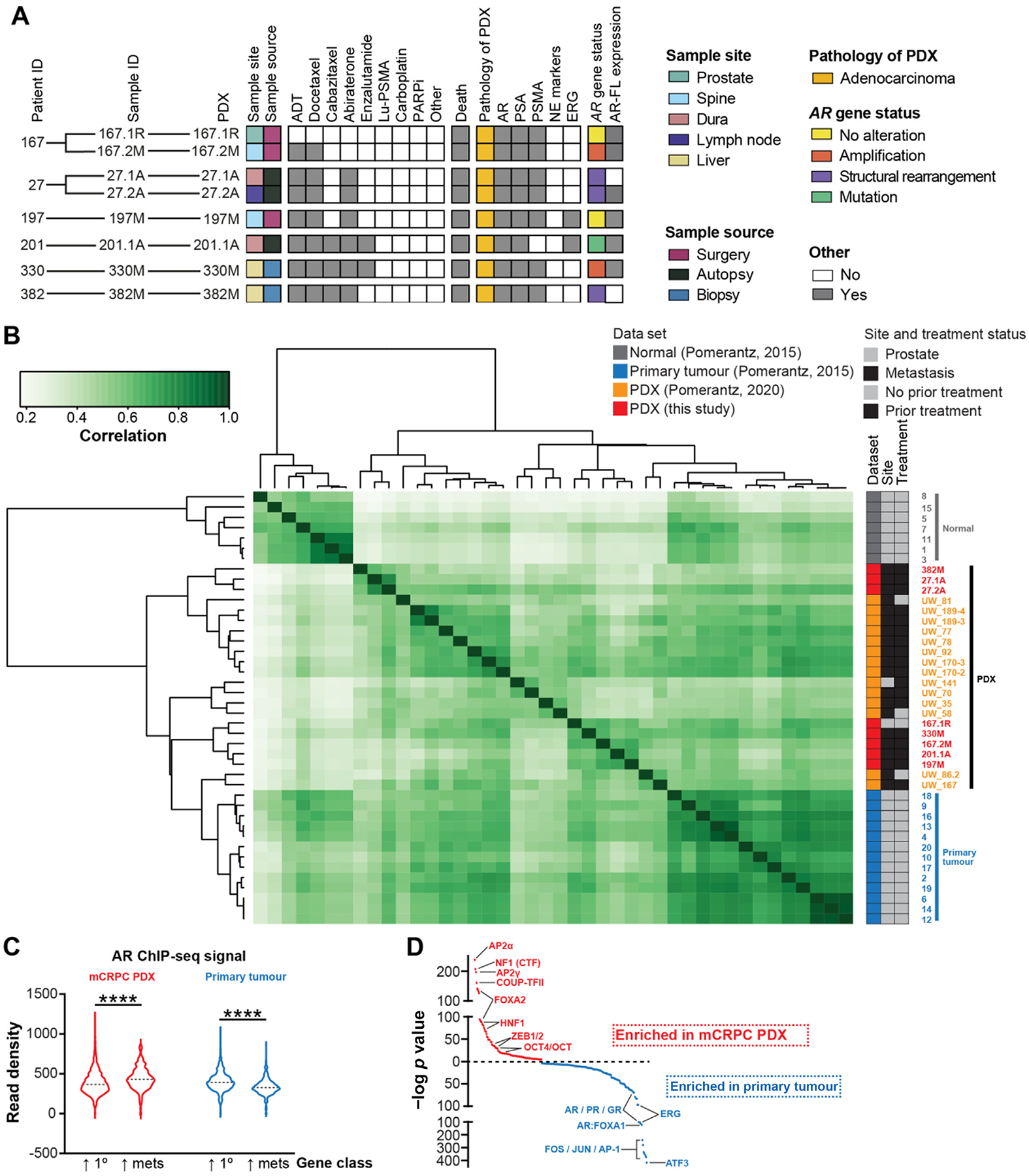
The AR has distinct transcriptional activity in metastatic prostate cancer. (A) Summary of the PDX cohort showing the origin of each PDX, the prior treatments the patients received before donating tissue, follow-up (death), the histopathology of the PDXs, and AR status. PDXs obtained from the same patient are indicated by branches between the patient and sample IDs. NE marker positivity denotes positive staining for at least one of chromogranin A, synaptophysin, and CD56; ERG represents ERG fusions. * PDX-201.1A has T878A and C687Y AR mutations. (B) PDXs have distinct AR cistromes in comparison to primary tumours and normal prostate tissue [19]. Clustering represents correlations between individual ChIP-seq samples using scores based on read counts for every sample (ie, affinity scores). The scale bar indicates Pearson correlation. (C) Genes upregulated in metastasis in comparison to primary tumours (↑ mets) exhibit a higher AR ChIP-seq signal (average AR read counts within 50 kb of gene transcriptional start sites) in PDXs of metastatic CRPC in comparison to primary tumours. By contrast, genes upregulated in primary tumours in comparison to metastasis (↑ 1°) exhibit a higher AR ChIP-seq signal in primary tumours than PDXs of metastatic CRPC (**** p < 0.0001; unpaired t tests). (D) Motif analysis of AR binding sites enriched in PDXs of metastatic CRPC or primary tumours. Enriched motifs are plotted according to their p value rank. ADT = androgen deprivation therapy; AR = androgen receptor; AR=FL = full-length AR; ChIP-seq = chromatin immunoprecipitation sequencing; CRPC = castration-resistant prostate cancer; mCRPC = metastatic CRPC; NE = neuroendocrine; PARPi = PARP inhibitor; PDX = patient-derived xenograft; PSA = prostate-specific antigen; PSMA = prostate-specific membrane antigen.

To interrogate oncogenic AR signalling, we analysed PDXs with diverse AR alterations (Fig. 1A) [11,12]. These PDXs were established from patients who had progressed on ADT and additional systemic treatments such as taxane chemotherapy and AR signalling inhibitors (Fig. 1A and Supplementary Fig. 1). We also included a PDX of a high-risk castrate-sensitive primary tumour (PDX-167.1R) from the same patient as PDX-167.2M, providing a patient-matched example of therapy-induced changes in AR signalling.

We used ChIP-seq to profile the genome-wide DNA binding profile (cistrome) of the AR in each PDX. The quality of the data sets was evident from detection of known AR binding sites (ARBSs; eg, proximal to the *GRHL2* gene; Supplementary Fig. 2A), the cistromic overlap rate (Supplementary Fig. 2B; *n* = 11 331 AR binding sites shared in ≥4/7 models), and the size of the cistromes (Supplementary Table 3; all >1900 peaks). Moreover, there was concordance across different PDX generations (Supplementary Fig. 2C). There was also a high degree of similarity between AR cistromes for PDX-27.1A and PDX-27.2A (Fig. 1B and Supplementary Fig. 2C), which represent distinct metastases from the same patient [12], further confirming the robustness of the data.

We compared our AR cistrome data to normal prostate tissues, primary tumours, and the LuCaP series of PDXs [19,20] and identified several clusters and subclusters (Fig. 1B). Normal prostate tissues were separated from all tumour samples. Patient primary tumours grouped together, as did PDXs, reflecting the combined impact of sample type, disease site, and treatment status. Notably, there was more heterogeneity among AR cistromes in PDXs than in primary patient tumours, with lower correlation coefficients between samples. This supports the concept that the AR cistrome is first transformed during tumourigenesis and is then further reprogrammed during cancer progression, with metastasis and treatment resistance increasing heterogeneity between tumours [20].

To further examine this idea, we analysed previously defined ARBS sets that distinguish normal (N-ARBS), tumour (T-ARBS), and metastatic (Met-ARBS) states [20]. As expected, there was minimal AR DNA binding to N-ARBS sites in the PDXs, but stronger enrichment for T-ARBS and Met-ARBS sites (Supplementary Fig. 2D). We identified common themes that support the functionality of altered AR binding in PDXs of metastatic CRPC versus treatment-naïve primary tumours. First, AR DNA binding was more strongly associated with genes upregulated in metastatic CRPC than in castration-sensitive primary tumours (Fig. 1C and Supplementary material) [21]. Second, AR binding events enriched in the metastatic PDXs compared to primary tumours were associated with DNA binding motifs of transcription factors linked to aggressive therapy-resistant phenotypes, such as ZEB1, COUP-TFII, and OCT4 (Fig. 1D) [22].

We also performed ChIP-seq for a histone marker of active enhancers and promoters, acetylated lysine 27 of histone H3 (H3K27ac). Mirroring the AR ChIP-seq data, the H3K27ac cistromes from PDXs of metastatic CRPC clustered separately from treatment-naive primary tumours [23] (Supplementary Fig. 3A). There was also more heterogeneity among the H3K27ac cistromes in the PDXs than in primary tumours according to lower average correlation coefficients. This highlights that transcriptional activity is reprogrammed during prostate cancer progression in a nonuniform manner, leading to greater heterogeneity in metastatic CRPC.

### 3.2. ARv567es has distinct activity from AR-FL

**Fig. 2 –:**
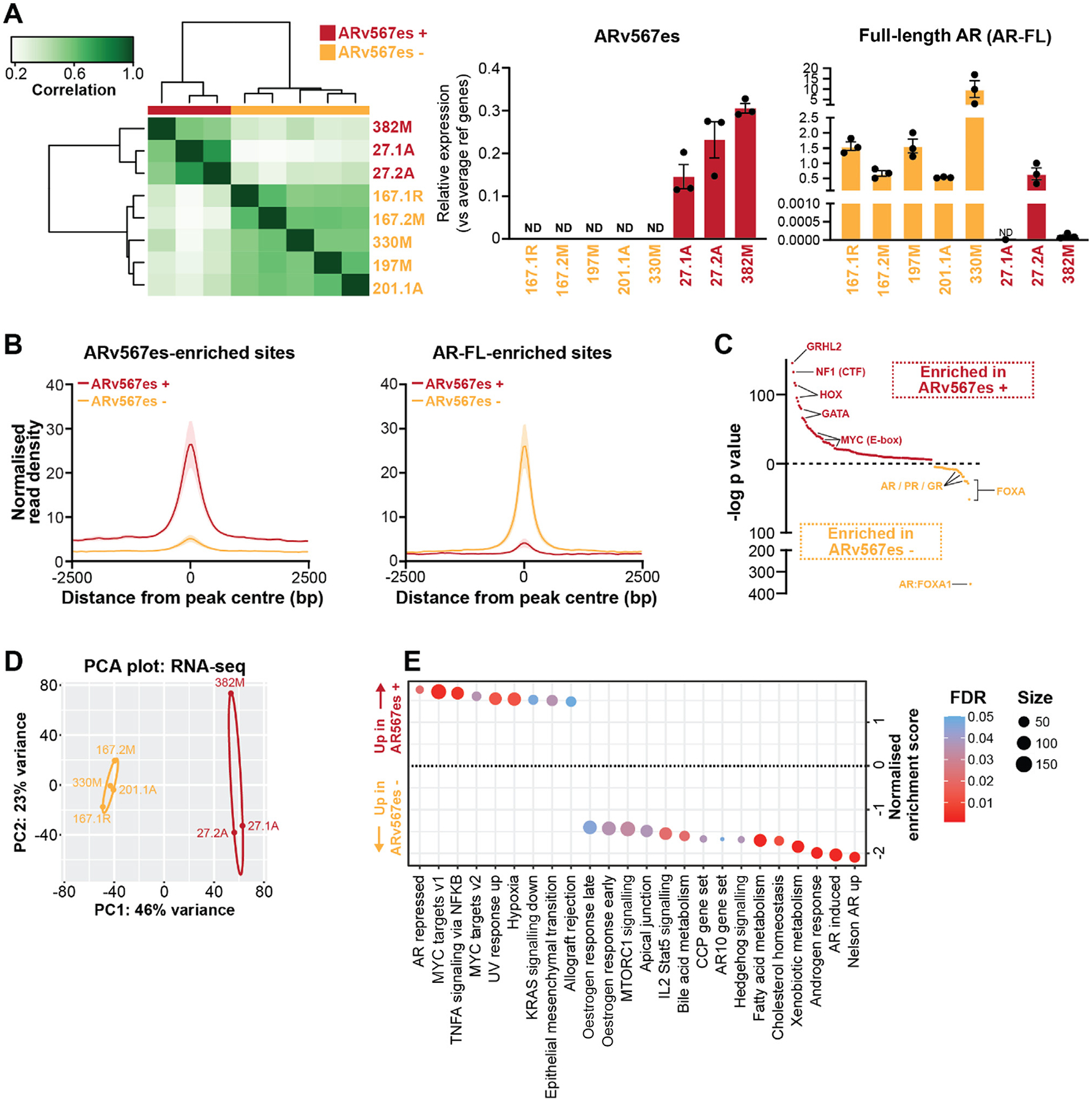
Comparison of ARv567es and AR-FL transcriptional activities in metastatic prostate cancer. (A) Left: AR cistromes from ARv567es-positive and - negative PDXs cluster separately. Clustering of the samples represents correlations between individual ChIP-seq samples according to scores based on read counts for every sample (ie, affinity scores). The scale bar indicates Pearson correlation. Right: Relative levels of AR-FL and ARv567es in the PDX models, as determined by quantitative reverse transcription-polymerase chain reaction normalised to the geometric mean for three reference genes. Error bars show ± the standard error of the mean. (B) Read density plots for AR ChIP-seq data proximal to ARv567es-enriched sites (left) and AR-FL-enriched sites (right). Data represent the average of the three ARv567es-positive and the five ARv567es-negative models. (C) Motif analysis of DNA sites enriched in ARv567es-positive versus ARv567es-negative PDXs. Enriched motifs are plotted according to their p value rank. (D) PCA plot of RNA-seq data showing clustering of ARv567es-positive and ARv567es-negative PDXs. (E) Normalised enrichment scores for gene sets that are significantly enriched (FDR < 0.05) according to relative gene expression levels in ARv567es-positive versus ARv567es-negative PDXs. AR = androgen receptor; AR-FL = full-length AR; CCP = cell-cycle proliferation; ChIP-seq = chromatin immunoprecipitation sequencing; FDR = false discovery rate; PCA = principal component analysis; PDX = patient-derived xenograft; RNA-seq = RNA sequencing.

PDXs 27.1A, 27.2A, and 382M formed a distinct cluster in the AR cistrome data (Fig. 1B). This clustering was sustained when considering only the PDXs in our series (Fig. 2A). These three PDXs also clustered according to their H3K27ac profiles (Supplementary Fig. 3B). Notably, these tumours all express ARv567es (Fig. 2A). This variant is associated with AR-GSRs that cause loss or exclusion of exons 5, 6, and 7 during transcription, resulting in an ARv567es protein lacking the ligand-binding domain [4,18,24–26]. Preclinical studies have shown that ARv567es is active in the absence of androgens and drives resistance to AR antagonists [24–26]. We confirmed our previous findings that PDX-27.1A almost entirely lacks the *AR-FL* transcript, which is only detectable at background levels (Fig. 2A). By contrast, PDX-27.2A expresses both *AR-FL* and *ARv567es* transcripts at comparable levels and probably represents a polyclonal AR-GSR (Fig. 2A and Supplementary Fig. 3C) [12]. Two targeted DNA sequencing approaches revealed that PDX-382M also contains an AR-GSR with deletion of exons 5–7 (Supplementary Fig. 3C,D); this genomic alteration results in exclusive expression of *ARv567es* mRNA and negligible expression of *AR-FL* mRNA (Fig. 2A).

ARv567es has been detected in patient samples and PDXs of CRPC [4,18,25]. While previous studies primarily examined ARv567es activity in cell lines with experimentally derived AR-GSRs [24,26], our patient-derived models provide an opportunity to interrogate the function of ARv567es in CRPC tissues. Therefore, we compared AR ChIP-seq data from the three ARv567es-positive PDXs to AR-v567es-negative models. We identified 1565 sites shared between the two groups, 1676 sites enriched in the ARv567es-positive PDXs, and 377 sites enriched in the AR-v567es-negative PDXs (Fig. 2B, Supplementary Fig. 4A, B, and Supplementary Table 4). The AR-FL-enriched binding sites closely resembled previously characterised AR and FOXA1 cistromes (Supplementary Fig. 4C) and had a higher frequency of AR and FOXA motifs (Fig. 2C). By contrast, ARv567es-enriched sites had a higher frequency of GRHL2, HOXA/B, GATA, and MYC motifs (Fig. 2C) and were associated with cistromes of these factors (Supplementary Fig. 4C).

RNA-seq of the PDXs further supported the hypothesis that ARv567es-postive tumours are transcriptionally distinct from ARv567es-negative tumours. The two groups formed distinct clusters (Fig. 2D). Pathways enriched in the ARv567es-positive PDXs included AR-repressed genes, MYC, and TNFα. ARv567es-negative PDXs were more strongly enriched for gene sets associated with AR-FL activity, such as androgen-regulated pathways (androgen response, Nelson AR up) and fatty acid/cholesterol metabolism (Fig. 2E). Collectively, these analyses indicate dampened canonical AR action in ARv567es-positive PDXs.

If ARv567es-enriched binding sites are functional, they could mediate altered transcription. To address this question, we identified genes predicted to be regulated by the ARv567es-enriched and AR-FL-enriched cistromes (Supplementary Table 5). Genes predicted to be regulated by ARv567es-enriched binding sites were upregulated in the ARv567es-positive PDXs; conversely, genes predicted to be regulated by AR-FL-enriched binding sites were upregulated in the ARv567es-negative PDXs (Supplementary Fig. 4D). We confirmed differences in the expression of candidate genes using qRT-PCR (Supplementary Fig. 4E,F).

We also compared ARv567es-positive PDXs from our cohort to independent models (LuCaP 86.2, which is predominantly ARv567es-positive; and LuCaP 136, which coexpresses ARv567es and AR-FL) [25,26]. PDXs clustered by patient and ARv567es status, which demonstrates that across cohorts, ARv567es-positive PDXs share transcriptional features (Supplementary Fig. 5A). Hierarchical clustering based on differentially expressed genes also grouped the tumours according to ARv567es status (Supplementary Fig. 5B).

Collectively, these integrated cistromic and transcriptomic analyses show that ARv567es and AR-FL have many shared AR binding sites and target genes, but that differences in their DNA binding profiles can mediate distinct transcriptional outcomes.

### 3.3. ARv567es expression is uncoupled from autoregulation

Next, we examined the relevance of ARv567es to therapeutic responses. We selected the two ARv567es-positive PDXs with and without AR-FL expression (PDX-27.1A, PDX-27.2A). The other ARv567es-positive PDX (PDX-382M) was excluded as the mice develop cachexia-like symptoms. For comparison, we also included two ARv567es-negative models with *AR* ligand-binding domain mutations (PDX-201.1A) [12] or *AR* amplification (PDX-167.2M) [11].

**Fig. 3 –:**
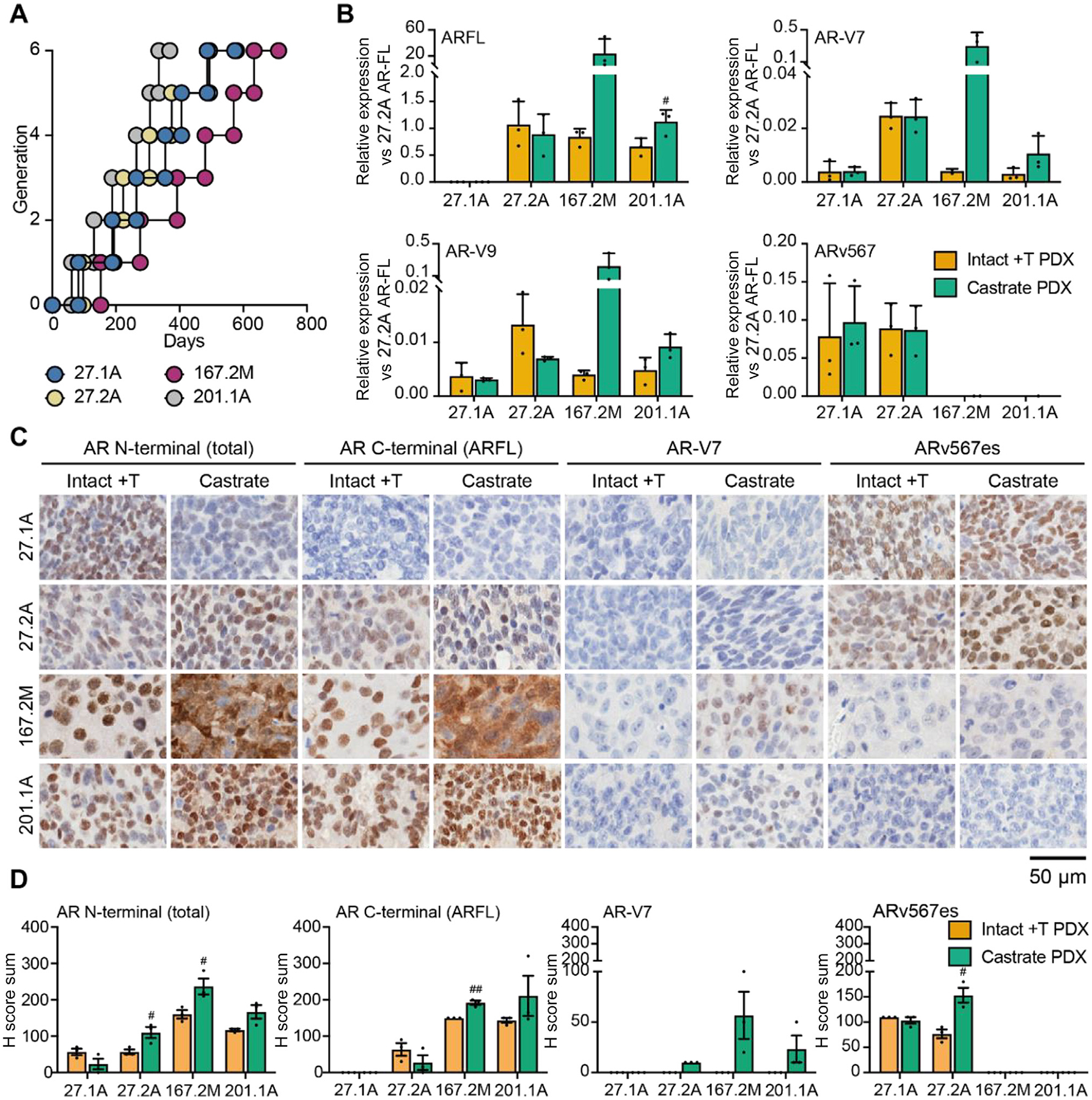
Response to castration in tumours with ARv567es. (A) Growth of PDXs 27.1A, 27.2A, 167.2M, and 201.1A for six generations after castration of host mice. Vertical lines (*y*-axis) indicate when PDXs reached the ethical cutoff of 1000 mm^3^ and were regrafted into castrated mice. The *x*-axis shows the number of days for each generation. (B) Quantitative reverse transcription-polymerase chain reaction data for AR-FL (exons 4–6), AR-V7, AR-V9, and ARv567es in each PDX for three generations before castration (intact +T PDX) and after castration (castrate PDX) of host mice (*n* = 3 generations per PDX per group). Data represent the relative expression of each transcript in comparison to AR-FL in 27.2A intact +T samples, which have all four amplicons. (C) Representative images of immunohistochemical staining for PDXs grown in intact + T or castrate mice. Primary antibodies were directed to the AR N-terminal domain (detecting total AR), AR C-terminal domain (detecting AR-FL), AR-V7, and ARv567es. The scale bar represents 50 μm. (D) Quantification of immunohistochemical staining for each antibody. Graphs show the average H score sum for nuclear and cytoplasmic staining for three generations before castration (intact + T) and after castration (castrate). Data in B and D are for three grafts per group. ^#^*p* < 0.05, ^##^*p* < 0.01 (higher for BAT vs vehicle; unpaired t tests). All error bars denote the standard error of the mean. AR = androgen receptor; AR-FL = full-length AR; PDX = patient-derived xenograft.

To assess responses to ADT, PDXs were initially grown in intact mice with testosterone implants before being castrated. The PDXs were then regrafted into castrated mice for subsequent generations. All four PDXs were castration-resistant according to observation of ongoing growth in castrated mice (Fig. 3A). Castration usually upregulates *AR* transcription as an adaptive response to low androgen levels [5]. To examine whether this occurred, we used qRT-PCR to measure the expression of *AR-FL*, represented by transcripts containing exons 4–6, and AR-Vs. As anticipated, castration increased *AR-FL* mRNA levels in PDXs lacking *ARv567es* expression and AR-GSRs (PDX-167.2M and PDX-201.1A). In these models, castration also upregulated *AR-V7* and *AR-V9*, which are isoforms that arise via alternative AR splicing rather than AR-GSRs (Fig. 3B). By contrast, *ARv567es* expression did not increase with castration in PDX-27.1A and PDX-27.2A, and *AR-FL* expression did not increase in PDX 27.2A (Fig. 3B). We confirmed this via IHC using antibodies detecting total AR, AR-FL, AR-V7, and ARv567es (Fig. 3C,D). Consistent with constitutively active AR-Vs, AR-V7 and ARv567es were predominantly localised to the nucleus, as expected [27,28]. By contrast, AR-FL was present in both the cytoplasm and the nucleus, especially in PDX-167.2M (Supplementary Fig. 6A). On castration, the results were largely consistent with changes in *AR* transcript levels. Thus, castration only increases *AR* expression in tumours without ARv567es. To expand these observations, we also treated PDX-27.2A, which expresses ARv567es and AR-FL, with enzalutamide. There was no change in tumour volume, confirming that this model is not only castration-resistant but also has a poor response to enzalutamide (Supplementary Fig. 6B).

**Fig. 4 –:**
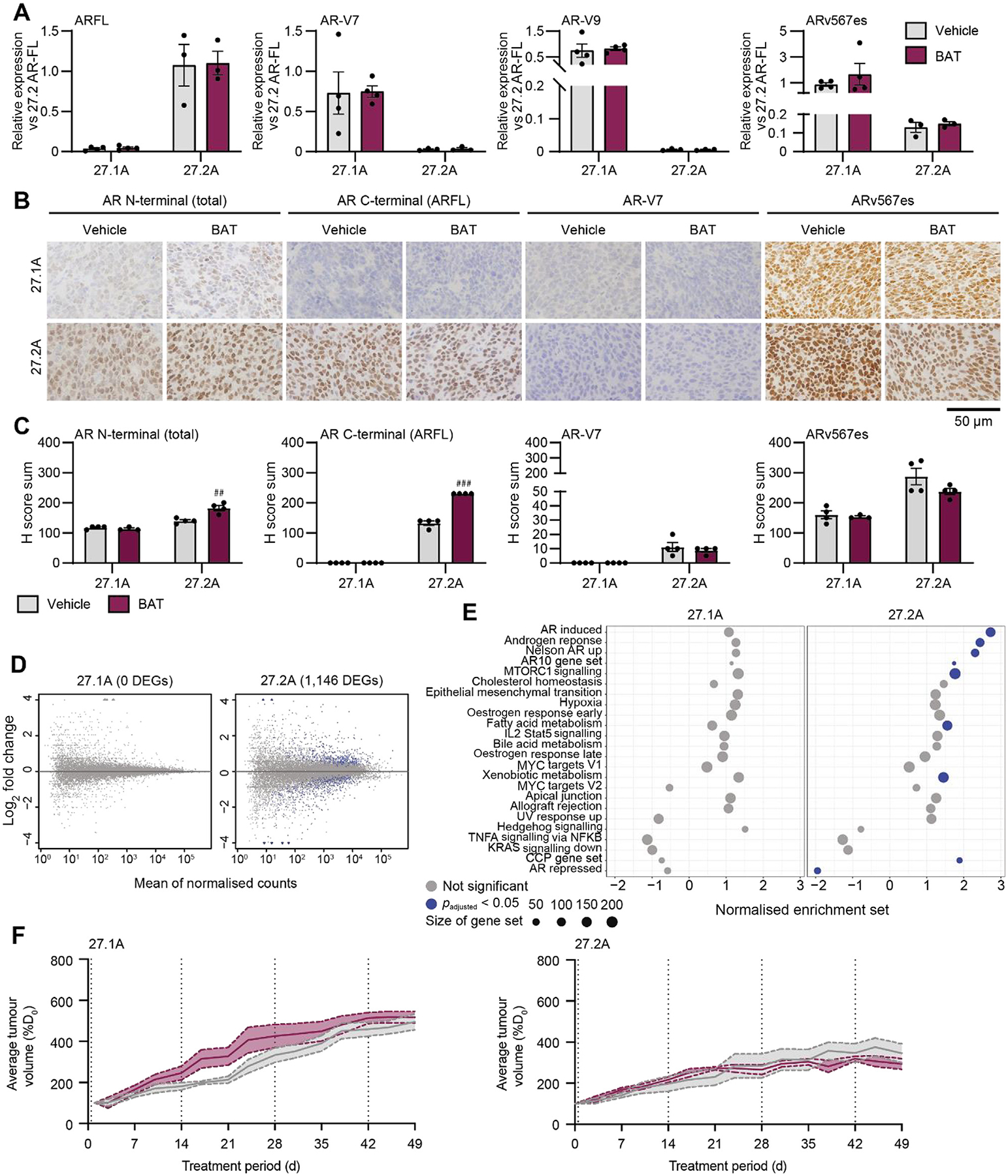
Response to BAT in tumours with ARv567es. (A) Quantitative reverse transcription-polymerase chain reaction data for AR-FL (exons 4–6), AR-V7, AR-V9, and ARv567es in PDXs treated for 24 h with vehicle control or testosterone cypionate. Data represents average expression relative to AR-FL in the PDX-27.2A vehicle control group (*n* = 3 per treatment group). (B) Representative images of PDXs after 24 h of treatment, stained using antibodies for the AR N-terminus (total AR), AR C-terminus (AR-FL), AR-V7, and ARv567es. The scale bar represents 50 μm. (C) Average H score sum for nuclear and cytoplasmic immunohistochemical staining of PDXs after 24 h of treatment. (D) Scatter plots showing the log_2_ fold change in mRNA abundance versus the mean counts for PDXs treated with control versus testosterone cypionate. Genes with significantly differential expression are shown in blue, and genes with nonsignificant differential expression in grey. (E) Plots of gene set enrichment analysis for BAT versus vehicle samples for each PDX. The same pathways as in Fig. 2F are shown. The size of each dot represents the number of genes in each gene set. Blue dots denote significant enrichment (adjusted *p* < 0.05); grey dots denote no significant change. (F) Changes in the average tumour volume relative to day 0 (%D_0_) for PDXs treated with vehicle control (grey) versus BAT (purple). The solid line represents the average volume and shaded areas represent the standard error of the mean (*n* = 7–8 per treatment group; *p = 0.002 for mixed-model analysis of treatment vs time). All error bars denote the standard error of the mean. Data in B and D are for three or four grafts per group. * *p* < 0.05, ** *p* < 0.01, *** *p* < 0.001, lower for BAT versus vehicle; ^##^ *p* < 0.01, ^###^ *p* < 0.001, higher for BAT versus vehicle (unpaired t tests). AR = androgen receptor; AR-FL = full-length AR; BAT = bipolar androgen therapy; CCP = cell-cycle proliferation; PDX = patient-derived xenograft.

BAT is an emerging treatment involving periodic administration of supraphysiological androgen doses [29]. BAT causes downregulation of the AR [30]. We hypothesised that ARv567es-positive tumours would be deficient in autoregulatory activity and resistant to BAT. First, we examined the impact of supraphysiological testosterone on AR levels after 1 d of treatment, when androgens are at their peak concentration. Supraphysiological testosterone treatment did not change *AR-FL* or AR-V mRNA levels in either ARv567es-positive tumour (Fig. 4A). We verified this at the protein level using IHC, which showed that supraphysiological testosterone caused little change or an increase in AR protein levels (Fig. 4B,C and Supplementary Fig. 6C). Thus, ARv567es-positive PDXs lack autoregulatory changes in AR expression in response to supraphysiological testosterone.

We investigated the transcriptional responses to treatment using RNA-Seq. Consistent with the lack of AR-FL in PDX-27.1A, there were no differentially expressed genes or pathways after testosterone treatment (Fig. 4D,E and Supplementary Table 6). PDX-27.2A, which expresses both ARv567es and AR-FL, had 1146 differentially expressed genes (Fig. 4E) and significant enrichment of AR-related pathways (eg, AR induced, androgen response, Nelson AR up; Fig. 4F). However, there was no decrease in cell-cycle proliferation or MYC gene sets, in contrast to results from previous studies [14,30]. Therefore, ARv567es-driven tumours have muted transcriptional responses to supraphysiological testosterone.

To determine the longer-term responses of ARv567es-positive PDXs to BAT, we treated mice with testosterone every 2 wks for 7 wks. Both ARv567es-positive PDXs were resistant to BAT, with no significant changes in tumour volume (Fig. 4A and Supplementary Table 7). Overall, this shows that ARv567es-driven tumours have poor responses to BAT according to evaluation of AR autoregulation, transcriptional changes, and tumour growth.

## 4. Discussion

The AR is a master transcription factor and oncogenic driver in prostate cancer, but questions remain regarding the relevance of truncated, constitutively active AR-Vs. Using a panel of PDXs, we showed that ARv567es reprograms AR activity and mediates resistance to AR-directed treatments. Therefore, genomic alterations that produce ARv567es impact treatment outcomes.

The involvement of AR-Vs is a rational mechanism for therapy resistance, but proving this concept has been challenging. For example, it has been reported that AR-V7 has altered chromatin binding [31,32], but interpretation is complicated by its coexpression and heterodimerisation with AR-FL [8,10,33]. Since we used PDXs with AR-GSRs, which isolates ARv567es expression from AR-FL, our study is an important advance. We showed that ARv567es can be expressed with (PDX-27.2A) or without (PDX-27.1A and PDX-382M) AR-FL. The former scenario is likely to be because of *AR* gene amplifications that permit the coexistence of intact *AR* gene copies alongside AR-GSRs [34]. We identified a set of AR binding sites that were enriched in ARv567es-positive tumours as well as binding sites that were shared among PDXs, regardless of their AR-FL and ARv567es levels. This warrants further studies into how the AR cistrome changes with treatment (including castration, AR signalling inhibitors, and BAT) in tumours with varying ARv567es and AR-FL expression.

We also demonstrated that ARv567es-positive tumours are resistant to BAT [29]. This is explained by AR-GSRs that preclude expression of AR-FL, which is required to respond to supraphysiological testosterone. To the best of our knowledge, all patient-derived models described to date with ARv567es and AR-GSRs are resistant to therapies targeting the AR ligand-binding domain [25]. This distinguishes ARv567es, which is due to a structural genomic alteration, from other AR-Vs, which arise via mRNA splicing and are thus strongly correlated with the *AR* copy number [35].

*AR* gene expression is usually modulated via an autoregulatory loop in which ligand-bound AR-FL interacts with its own gene to repress transcription [36]. BAT exploits this loop to mitigate resistance to continuous low and high AR activity [29]. Castration and BAT had little impact on ARv567es levels, demonstrating that AR-mediated gene autoregulation was disrupted. Future studies could use additional models to examine the impact of different genomic structural variants on AR autoregulation. Beyond AR levels, ARv567es-positive tumours had minimal transcriptional responses to supraphysiological testosterone. This identifies the mechanism underpinning the resistance of tumours with high ARv567es expression to AR-directed therapies.

ARv567es is a potential predictive biomarker; however, its precise frequency in CRPC is uncertain. Tissue-based analyses suggest that ARv567es is present in approximately 5% of advanced prostate cancers [4,11,18]. By contrast, analyses of blood and circulating tumour cells have detected ARv567es or AR-GSRs that truncate the ligand-binding domain of AR in 4–78% of patients [37–40]. This variation may be due to differences in cohorts and methods, so more widespread analyses are needed. Notably, detection of ARv567es or AR-GRSs in liquid biopsies has been linked to disease state, treatment responses, and progression-free survival [37–40]. Therefore, ARv567es could be used to guide treatment and monitor patients for signs of resistance to AR-directed therapies. Patients with ARv567es-high tumours could be prioritised for non–AR-directed therapies (eg, radioligand therapy targeting prostate-specific membrane antigen) or emerging treatments that disrupt AR activity in different ways, such as inhibitors of the N-terminal domain or compounds that block chromatin remodelling (eg, CBP/p300, LSD1) [41–43].

## 5. Conclusions

The trajectory of AR transcriptional reprogramming during progression to CRPC is influenced by ARv567es, an important truncated AR variant arising from AR-GSRs. ARv567es should be further evaluated as a therapeutic target and predictive biomarker. More broadly, future studies should profile the AR cistrome in large CRPC cohorts to subclassify the spectrum of AR activity across different mechanisms of resistance and relate it to treatment responses.

## Supplementary Material

1

2

3

## Data Availability

Data are available on request via dbGaP (https://www.ncbi.nlm.nih.gov/gap; phs003369.v3.p1).

## ADVANCING PRACTICE

### What does this study add?

The role of androgen receptor (AR) variants as mediators of therapy resistance remains controversial. Our study addressed this knowledge gap using contemporary patient-derived models in which the expression of AR variants (ARv567es) is uncoupled from full-length AR because of gene rearrangements. We showed that this results in reprogramming of AR activation and disruption of AR autoregulation, and that ARv567es-positive tumours are resistant to androgen deprivation and bipolar androgen therapy. Therefore, ARv567es should be further evaluated as a therapeutic target and predictive biomarker.

### Clinical Relevance

This study shows that ARv567es drives resistance to hormone-based therapies in metastatic prostate cancer. ARv567es may serve as a predictive biomarker and guide alternative treatment strategies.

### Patient Summary

We compared different ways in which prostate cancer cells adapt to treatment. In a subset of tumours, we found disruption of hormone responses that promotes resistance to therapy. Tumours with these features are not suitable for standard hormone-based treatments.

## Appendix A. Supplementary data

Supplementary data to this article can be found online at https://doi.org/10.1016/j.euf.2025.03.017.
