# Supplementary material for: Reprogramming of Androgen Receptor Activity in Castration-resistant Prostate Cancer is Shaped by Truncated Variants
Source: Eur Urol Focus. Author manuscript; Available in PMC 2026 May 17. (PMC13180466; doi:10.1016/j.euf.2025.03.017)
Supplement: 1 [file NIHMS2174467-supplement-1.docx]

**SUPPLEMENTARY METHODS**

**Acquisition of patient specimens**

Written informed consent was obtained from patients according to institutional human ethics approvals from Monash University (1636; 36762; 12287), the Peter MacCallum Cancer Centre (11/102), Cabrini Hospital (03-14-04-08) and Eastern Health (E55/1213). Prostate cancer tissues were collected from: 1) localised prostate cancer specimens from patients undergoing radical prostatectomy; 2) CRPC specimens collected during biopsies of metastases; and 3) CRPC samples from rapid autopsies.

**Xenografting**

PDXs were previously established by the Melbourne Urological Research Alliance (MURAL)^1-3^. All animal care complied with Monash University animal ethics approvals (MARP 2012/158, MARP 2014/085, MARP 22185 and MARP 28911). PDXs were established by grafting up to 3 pieces of tumour tissue (approximately 4 mm^3^) under each renal capsule of 6–8-week-old male NOD-SCID or NSG mice. The mice were supplemented with 5 mm testosterone implants to improve tumour take rate, as previously described^4,5^. Mice were palpated at least twice per week for signs of growth. Actively growing grafts were then transplanted to new host mice with testosterone implants. Regrafting was not performed if there was no residual tumour tissue or if host mice developed lymphoma. After two or three generations, some serially transplantable PDXs were regrafted subcutaneously (approximately 9 mm^3^) for subsequent generations. Serially transplantable PDXs were regrafted into new host mice every ~10 weeks (range 4-30 weeks) before they reached a maximum volume of 1000 mm^3^. In subsequent generations (see PDX treatments) CRPC PDXs were transferred to castrated hosts. PDXs were routinely authenticated by profiling short tandem repeats, and confirming the lack of lymphoma using immunohistochemistry for CD45.

**Chromatin immunoprecipitation sequencing (ChIP-seq)**

Early generation (≤10) PDXs grown as subcutaneous or subrenal grafts in intact mice with testosterone implants were collected at endpoint. The tumours were cryopreserved in OCT compound (Tissue-Tek), and sectioned, cross-linked, and processed for ChIP as described^6^. To examine biological variation, we analysed samples of tissue from different generations for PDXs 167.1R, 27.1A and 27.2A. Chromatin was obtained from the equivalent of 50 x 30 μm slices (AR) or 20 x 30 μm slices (H3K27ac). For AR, we used a pool of two antibodies for each ChIP: Abcam 74272 (3 µg) and Millipore PG-21 (2 µg). For H3K27ac, we used 2 µg of Abcam 4729 per ChIP. ChIPs were performed as described^6^ and the resultant DNA was prepared in 20 µl of nuclease-free water. Libraries were prepared from 10 ng of DNA using an Ultra Low Library Preparation Kit (Qiagen), according to the manufacturer’s protocol. Prepared libraries were sequenced on the NextSeq OH (Illumina) with single-end 75 bp sequencing at the South Australian Genomics Centre, targeting a depth of ≥25 million reads per sample.

Raw sequencing data was processed and mapped to human assembly GRCh37 (hg19) as described previously^7^. Peaks were called using MACS2^8^ against a pooled input sample using default parameters except for applying an FDR cutoff of 0.01. Bigwigs were generated using Deeptools^9^ and visualised with Integrative Genomics Viewer^10^. Read density plots, heatmaps and bigwig correlation plots were generated using deepTools. Raw (i.e. fastq) published AR ChIP-seq data derived from primary tumours^11^ and LuCaP PDXs^12^ was downloaded from the Sequence Read Archive (SRA) using the SRA-Toolkit (version 2.5.4) and processed as described above to allow direct comparison. Raw (i.e. fastq) published H3K27ac ChIP-seq data derived from primary tumours ^13^ was downloaded from the European Genome-Phenome Archive (EGAD00001003461) after obtaining approval for access from the Blueprint Epigenome Consortium. Clustering of the MURAL PDX data and the previously published data was done using DiffBind^14^ in R 3.6.2 using peaks called with MACS2; for PDXs with ChIP-seq data from multiple replicates/passages (27.1A, 27.2A and 1671R), peaks called from merged replicate data were used. The cistrome overlap rate for the MURAL PDXs was determined using DiffBind. To identify ARv567es-enriched and AR-FL-enriched peaks, we used DiffBind to generate consensus peak sets for the ARv567es-positive and ARv567es-negative groups (minOverlap = 0.8 i.e. consensus peaks were in 3/3 or 4/5 models for the ARv567es-positive and ARv567es-negative groups, respectively); any peaks that were present in the other group were subsequently removed to yield the ARv567es-enriched and AR-FL-enriched peak sets. GIGGLE plots were generated using the Cistrome data browser^15^. Differential motif analysis was determined for cistromes of interest using the HOMER^16^ findmotifsgenome.pl function with the opposing dataset set as background (-size 200). To evaluate AR binding at genes associated with primary or metastatic disease, mRNA expression data from the Taylor dataset^17^ comprising 131 primary tumours and 31 metastases was downloaded from cBioPortal^18^ and transcripts differentially expressed between the two groups were identified (adjusted P ≤ 0.01). Read density within 50 kb of the transcriptional start sites of these genes was quantified using HOMER annotatePeaks.pl function.

Genes associated with ARv567es-enriched and AR-FL-enriched peaks were identified using BETA^19^; these gene sets were integrated with transcriptomic data using Gene Set Enrichment Analysis^20^.

**Quantitative RT-PCR**

Total RNA from PDXs was isolated using the RNeasy Kit (QIAGEN) with an on-column DNase-I treatment according to the manufacturer’s instructions. Gene expression was examined by RT-PCR performed on samples using Power SYBR Green Master Mix (ThermoFisher Scientific) or TaqMan RT-PCR Assay (ThermoFisher Scientific) and Mx3000 RT-PCR Software (Agilent Technologies). The relative mRNA expression of target genes was calculated using the ΔΔCt method and normalised against the geometric mean of the *RPLPO*, *TMEM199* and *ZNF207* reference genes as previously described^2^. Primer sequences are listed in Supplementary Table 2, as previously described^21^.

***AR*-targeted DNA sequencing and data analysis**

Genomic DNA was isolated from PDXs and analysed by a custom *AR*-targeted DNA-sequencing assay as described^22,23^. *AR* gene structural rearrangements (*AR*-GSRs) were identified by the consensus of two independent structural variant callers (LUMPY and Delly) as described. BAM files of unique (duplicates removed) mapped reads were visualized in Integrative Genomics Viewer (IGV)^24^.

**Treatment of PDXs**

To model androgen deprivation therapy, subcutaneous grafts were established in testosterone-supplemented host mice until tumour volume reached ~200 mm^3^. At this point, host mice were castrated and their testosterone pellet removed. To assess tumour growth over subsequent generations, PDXs were regrafted directly into castrated host mice.

For enzalutamide treatment, PDX-27.2A was established as subcutaneous grafts in castrated mice (1 graft/mouse). Once grafts were 100-200 mm^3^, mice were systematically allocated to receive vehicle (5% DMSO, 0.95% carboxymethycellulose, 0.1% Tween 80), 10 mg/mg enzalutamide (SelleckChem) or 60 mg/kg enzalutamide. Mice were treated by gavage for five days per week and tumour volumes were measured three times per week with callipers.

To model bipolar androgen therapy, PDXs were established subcutaneously in castrated mice (1 graft/mouse) and maintained until tumour volume reached 100-200 mm^3^. Mice were then systematically assigned to either vehicle or testosterone treatment. For this, mice were treated with vehicle or 1 mg testosterone cypionate dissolved in 95% sesame oil + 5% ethanol via intramuscular injection every two weeks, as previously described^25^. Mice were treated every 14 days for up to 7 weeks, and tumour volume was measured using callipers three times per week throughout the treatment period. If tumours reached the maximum ethical volume of 1000 mm^3^, treatment was terminated early and tumours were collected to comply with animal ethics approvals.

**Immunohistochemistry**

Immunohistochemistry staining was performed on 5 µm-thick sections of formalin-fixed paraffin-embedded PDX tissue using the Leica BOND-MAX automated system (Leica Biosystems). Antibody details and staining conditions are listed in Supplementary Table 1. Slides were imaged using the Aperio ScanScope AT Turbo slide scanner (Aperio). Nuclear and cytoplasmic expression of AR protein was detected using specific antibodies to the AR N-terminal, AR C-terminal, AR-V7 and ARv567es. Staining was assessed using the semiquantitative modified H-score method. H-scores were calculated using the formula: [(% of no staining) × 0] + [(% of weak staining) × 1] + [(% of moderate staining) × 2] + [(% of strong staining) × 3]^26^. Nuclear and cytoplasmic staining were scored separately and the sum of both H scores was used to compare overall changes in staining.

**RNA sequencing and data analysis**

Total RNA was isolated using Qiagen RNeasy kits with on-column DNase-I treatment, and checked for purity and integrity with an Agilent TapeStation 4200 (Agilent Technologies). RNA sequencing was done at the Molecular Genomics Core, Peter MacCallum Cancer Centre. Libraries were prepared using the NEB stranded protocol and sequenced using NextSeq HO with 75 bp paired-end reads at the Australian Genome Research Facility. FastQC v0.11.6 was used to check the quality of raw reads. Cutadapt v2.1 was used to trim low-quality bases. STAR aligner v2.7.5b was used to align trimmed reads to the reference human (hg38) and mouse (mm39) genomes separately. Subsequently, XenofilteR v1.6 was used to select human specific reads and HTseq v0.11.2 was used to generate a counts matrix. DESeq2^27^ was used to read the count matrix and transformed further using variance stabilizing transformation (VST) function. The effect of batch on the transformed data was removed using removeBatchEffect from limma v3.52.1 package^28^. The scatter plot showing the log2 fold changes across the mean of normalized counts for each PDX sample was depicted using the plotMA function in DESeq2 v1.36.0. For gene set enrichment analyses, all the genes were ranked by log2foldchange and tested for the enrichment of Human Molecular Signatures Database (MSigDB)^20^ 50 cancer hallmarks pathways using the fgsea v1.22.0 R package. The dot plot of significantly (FDR<=0.05) enriched pathway terms were depicted using ggplot2 in R v4.2.0. The differentially expressed genes between ARv567es-positive versus AR-v567es-negative MURAL and LuCaP PDXs were further filtered based on FDR <0.01, logFC ≥2 or logFC ≤-2). Single sample gene set enrichment analysis of 50 hallmark gene sets in PDXs in response to BAT were calculated using the GSVA R package^29^. Differentially expressed genes and gene set enrichment analysis scores were depicted in heatmaps using the pheatmap v1.0.12 R package.

**Data availability**

The following datasets were accessed for this study: Gene Expression Omnibus datasets GSE56288 and GSE130408; and European Genome-Phenome Archive dataset EGAD00001003461. The ChIP-seq and RNA-seq data generated in this study are available upon request via the database of Genotypes and Phenotypes (dbGaP) at <https://www.ncbi.nlm.nih.gov/gap/> (study identification number phs003369.v3.p1).

**References**

1. Risbridger GP, Clark AK, Porter LH, et al. The MURAL collection of prostate cancer patient-derived xenografts enables discovery through preclinical models of uro-oncology. *Nat Commun*. Aug 19 2021;12(1):5049. doi:10.1038/s41467-021-25175-5

2. Lawrence MG, Obinata D, Sandhu S, et al. Patient-derived Models of Abiraterone- and Enzalutamide-resistant Prostate Cancer Reveal Sensitivity to Ribosome-directed Therapy. *Eur Urol*. Nov 2018;74(5):562-572. doi:10.1016/j.eururo.2018.06.020

3. Porter LH, Bakshi A, Pook D, et al. Androgen receptor enhancer amplification in matched patient-derived xenografts of primary and castrate-resistant prostate cancer. *J Pathol*. Jun 2021;254(2):121-134. doi:10.1002/path.5652

4. Toivanen R, Frydenberg M, Murphy D, et al. A preclinical xenograft model identifies castration-tolerant cancer-repopulating cells in localized prostate tumors. *Sci Transl Med*. May 29 2013;5(187):187ra71. doi:10.1126/scitranslmed.3005688

5. Lawrence MG, Taylor RA, Toivanen R, et al. A preclinical xenograft model of prostate cancer using human tumors. *Nat Protoc*. May 2013;8(5):836-48. doi:10.1038/nprot.2013.043

6. Singh AA, Schuurman K, Nevedomskaya E, et al. Optimized ChIP-seq method facilitates transcription factor profiling in human tumors. *Life Sci Alliance*. Feb 2019;2(1):e201800115. doi:10.26508/lsa.201800115

7. Hickey TE, Selth LA, Chia KM, et al. The androgen receptor is a tumor suppressor in estrogen receptor-positive breast cancer. *Nat Med*. Feb 2021;27(2):310-320. doi:10.1038/s41591-020-01168-7

8. Feng J, Liu T, Qin B, Zhang Y, Liu XS. Identifying ChIP-seq enrichment using MACS. *Nat Protoc*. Sep 2012;7(9):1728-40. doi:10.1038/nprot.2012.101

9. Ramirez F, Ryan DP, Gruning B, et al. deepTools2: a next generation web server for deep-sequencing data analysis. *Nucleic Acids Res*. Jul 8 2016;44(W1):W160-5. doi:10.1093/nar/gkw257

10. Robinson JT, Thorvaldsdottir H, Winckler W, et al. Integrative genomics viewer. *Nat Biotechnol*. Jan 2011;29(1):24-6. doi:10.1038/nbt.1754

11. Pomerantz MM, Li F, Takeda DY, et al. The androgen receptor cistrome is extensively reprogrammed in human prostate tumorigenesis. *Nat Genet*. Nov 2015;47(11):1346-51. doi:10.1038/ng.3419

12. Pomerantz MM, Qiu X, Zhu Y, et al. Prostate cancer reactivates developmental epigenomic programs during metastatic progression. *Nat Genet*. Aug 2020;52(8):790-799. doi:10.1038/s41588-020-0664-8

13. Kron KJ, Murison A, Zhou S, et al. TMPRSS2-ERG fusion co-opts master transcription factors and activates NOTCH signaling in primary prostate cancer. *Nat Genet*. Sep 2017;49(9):1336-1345. doi:10.1038/ng.3930

14. Ross-Innes CS, Stark R, Teschendorff AE, et al. Differential oestrogen receptor binding is associated with clinical outcome in breast cancer. *Nature*. Jan 4 2012;481(7381):389-93. doi:10.1038/nature10730

15. Zheng R, Wan C, Mei S, et al. Cistrome Data Browser: expanded datasets and new tools for gene regulatory analysis. *Nucleic Acids Res*. Jan 8 2019;47(D1):D729-D735. doi:10.1093/nar/gky1094

16. Heinz S, Benner C, Spann N, et al. Simple combinations of lineage-determining transcription factors prime cis-regulatory elements required for macrophage and B cell identities. *Mol Cell*. May 28 2010;38(4):576-89. doi:10.1016/j.molcel.2010.05.004

17. Taylor BS, Schultz N, Hieronymus H, et al. Integrative genomic profiling of human prostate cancer. *Cancer Cell*. Jul 13 2010;18(1):11-22. doi:10.1016/j.ccr.2010.05.026

18. Cerami E, Gao J, Dogrusoz U, et al. The cBio cancer genomics portal: an open platform for exploring multidimensional cancer genomics data. *Cancer Discov*. May 2012;2(5):401-4. doi:10.1158/2159-8290.CD-12-0095

19. Wang S, Sun H, Ma J, et al. Target analysis by integration of transcriptome and ChIP-seq data with BETA. *Nat Protoc*. Dec 2013;8(12):2502-15. doi:10.1038/nprot.2013.150

20. Subramanian A, Tamayo P, Mootha VK, et al. Gene set enrichment analysis: a knowledge-based approach for interpreting genome-wide expression profiles. *Proc Natl Acad Sci U S A*. Oct 25 2005;102(43):15545-50. doi:10.1073/pnas.0506580102

21. Gillis JL, Selth LA, Centenera MM, et al. Constitutively-active androgen receptor variants function independently of the HSP90 chaperone but do not confer resistance to HSP90 inhibitors. *Oncotarget*. May 2013;4(5):691-704. doi:10.18632/oncotarget.975

22. Li Y, Yang R, Henzler CM, et al. Diverse AR Gene Rearrangements Mediate Resistance to Androgen Receptor Inhibitors in Metastatic Prostate Cancer. *Clin Cancer Res*. Apr 15 2020;26(8):1965-1976. doi:10.1158/1078-0432.CCR-19-3023

23. Henzler C, Li Y, Yang R, et al. Truncation and constitutive activation of the androgen receptor by diverse genomic rearrangements in prostate cancer. *Nat Commun*. Nov 29 2016;7:13668. doi:10.1038/ncomms13668

24. Thorvaldsdottir H, Robinson JT, Mesirov JP. Integrative Genomics Viewer (IGV): high-performance genomics data visualization and exploration. *Brief Bioinform*. Mar 2013;14(2):178-92. doi:10.1093/bib/bbs017

25. Lam HM, Nguyen HM, Labrecque MP, et al. Durable Response of Enzalutamide-resistant Prostate Cancer to Supraphysiological Testosterone Is Associated with a Multifaceted Growth Suppression and Impaired DNA Damage Response Transcriptomic Program in Patient-derived Xenografts. *Eur Urol*. Feb 2020;77(2):144-155. doi:10.1016/j.eururo.2019.05.042

26. Detre S, Saclani Jotti G, Dowsett M. A "quickscore" method for immunohistochemical semiquantitation: validation for oestrogen receptor in breast carcinomas. *J Clin Pathol*. Sep 1995;48(9):876-8. doi:10.1136/jcp.48.9.876

27. Love MI, Huber W, Anders S. Moderated estimation of fold change and dispersion for RNA-seq data with DESeq2. *Genome Biol*. 2014;15(12):550. doi:10.1186/s13059-014-0550-8

28. Ritchie ME, Phipson B, Wu D, et al. limma powers differential expression analyses for RNA-sequencing and microarray studies. *Nucleic Acids Res*. Apr 20 2015;43(7):e47. doi:10.1093/nar/gkv007

29. Hanzelmann S, Castelo R, Guinney J. GSVA: gene set variation analysis for microarray and RNA-seq data. *BMC Bioinformatics*. Jan 16 2013;14:7. doi:10.1186/1471-2105-14-7
