# Supplementary material for: Reprogramming of Androgen Receptor Activity in Castration-resistant Prostate Cancer is Shaped by Truncated Variants
Source: Eur Urol Focus. Author manuscript; Available in PMC 2026 May 17. (PMC13180466; doi:10.1016/j.euf.2025.03.017)
Supplement: 3 [file NIHMS2174467-supplement-3.docx]

**Supplementary figures**

**
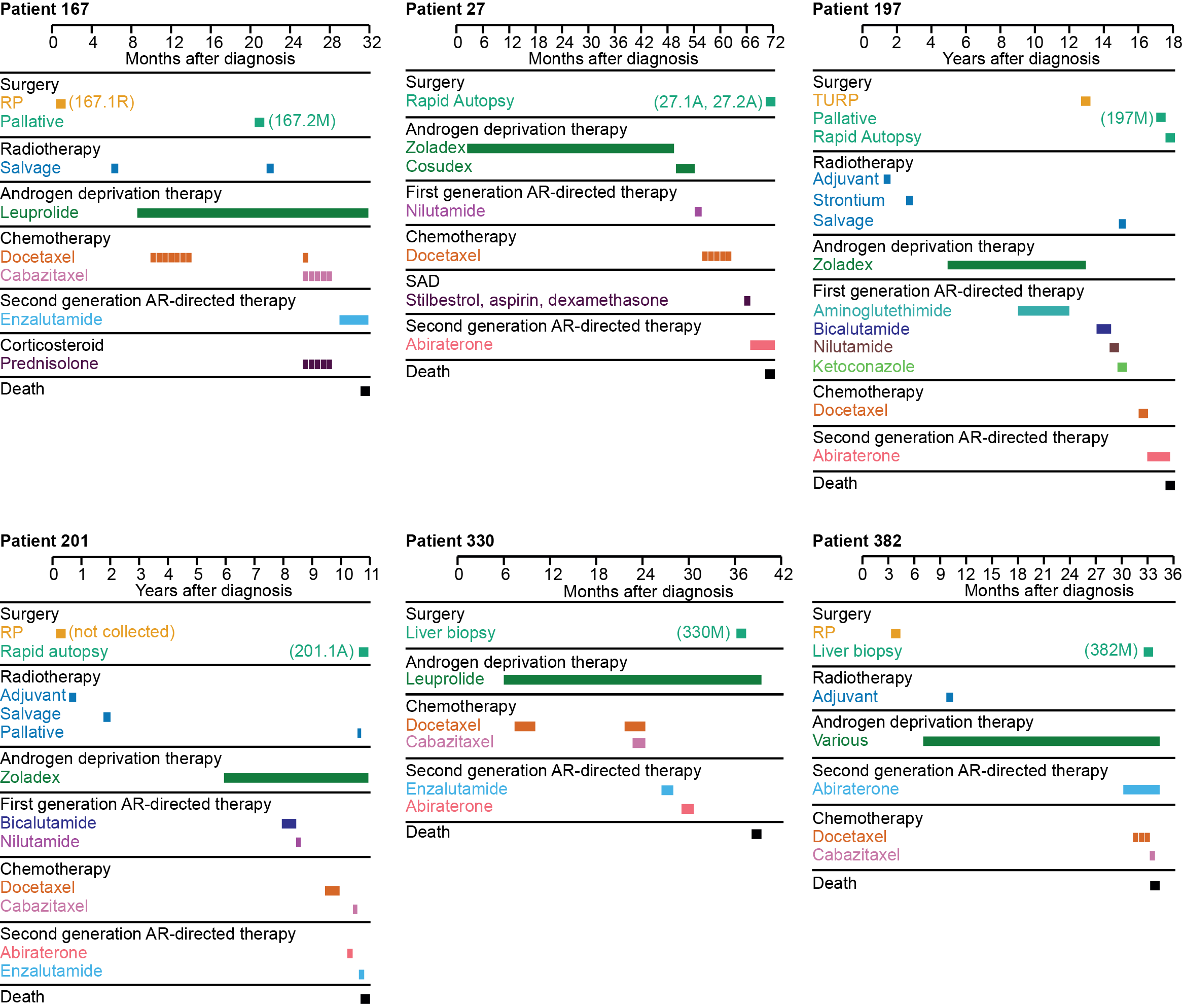
**

**Supplementary Figure 1. Patient treatment histories.** Diagrams summarising the timeline of treatment for each patient that donated tissue for PDXs in this study. Each timeline begins at the point of diagnosis. The coloured bands show the timing of each treatment, when tissue was collected for xenografting, and when patients died from prostate cancer.


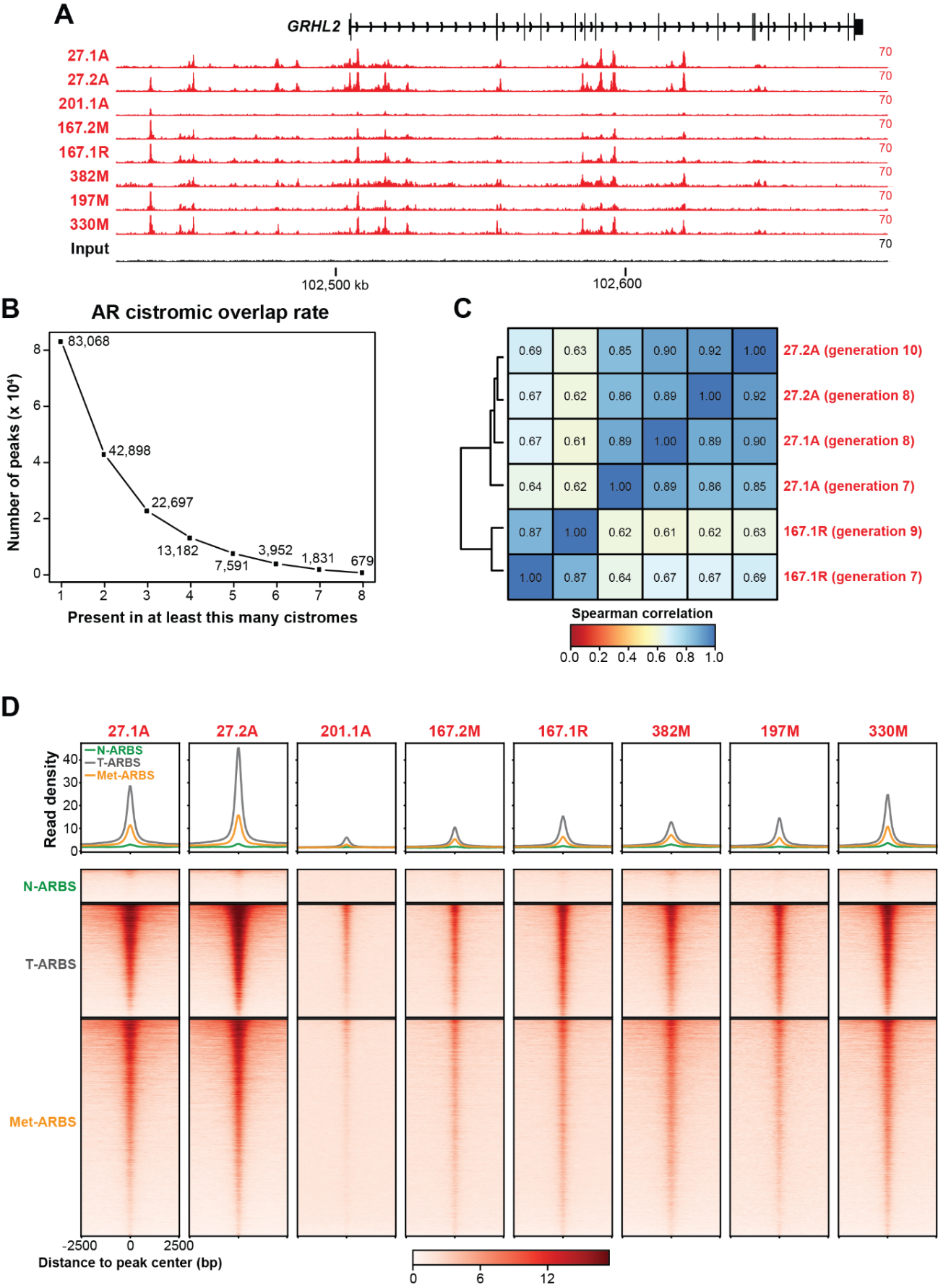


**Supplementary Figure 2. AR cistromes in prostate cancer PDXs. (A)** Genome browser images showing PDX AR ChIP-seq signals proximal to the *GRHL2* gene. **(B)** Plot showing the number of peaks that are shared across different numbers of PDXs (AR cistrome overlap rate). A large number of peaks were detected in a single PDX, while 679 peaks were detected across 8 PDXs. **(C)** Correlation between AR ChIP-seq data from different PDX generations. Spearman correlation values are shown. **(D)** Heat maps indicating AR ChIP-seq signal intensity in the PDXs at sites associated with normal AR binding (N-ARBS), primary tumour AR binding (T-ARBS) and metastatic tumour AR binding (Met-ARBS) ^20^.


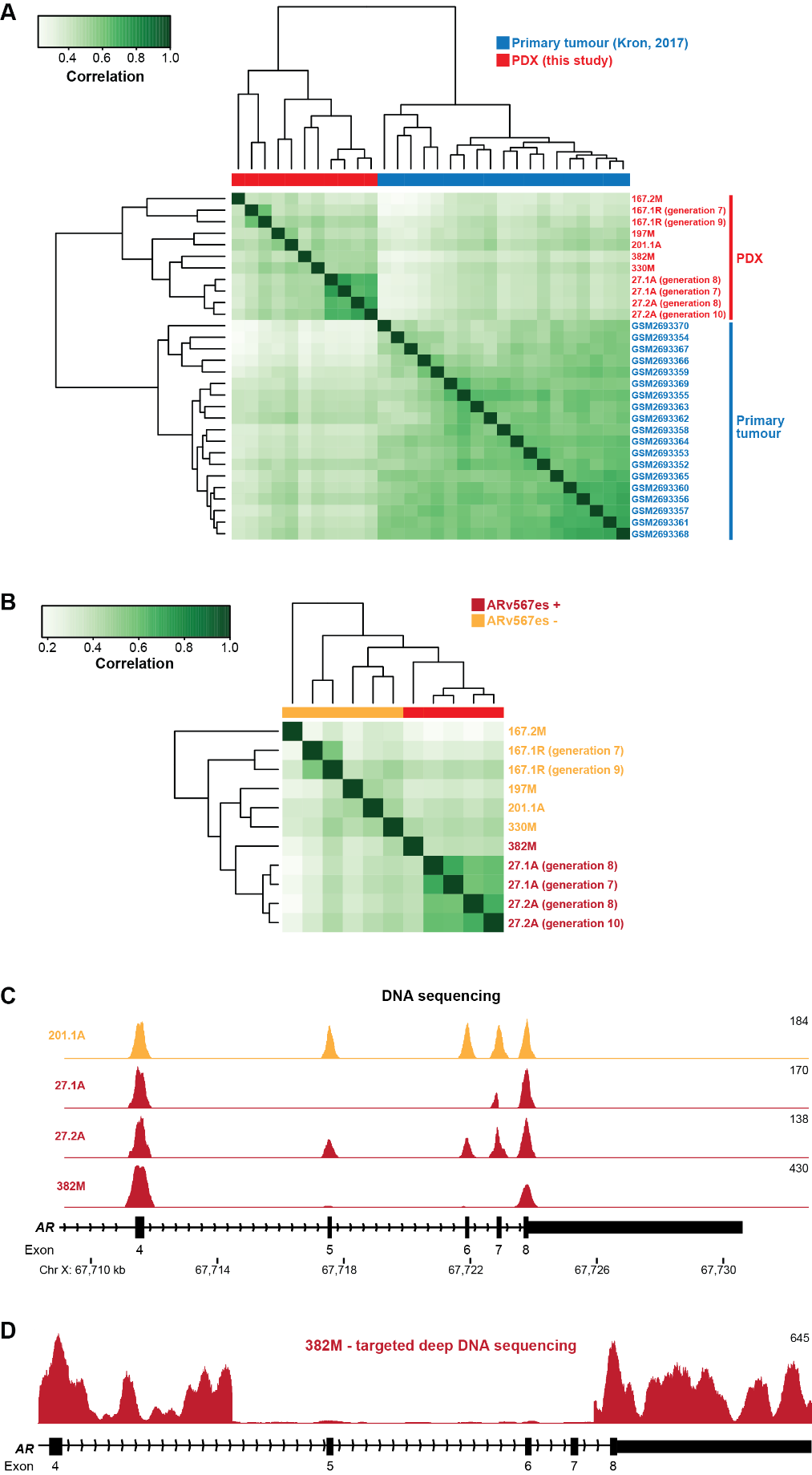


**Supplementary Figure 3. H3K27ac landscapes are altered in metastatic prostate cancer. (A)** PDX H3K27ac cistromes are distinct from primary tumours. Clustering represents correlations between individual ChIP-seq samples using scores based on read counts for every sample (i.e. affinity scores). Scale bar indicates Pearson correlation. **(B)** H3K27ac cistromes from ARv567es-positive and -negative PDXs cluster separately. Clustering is as described in (A). **(C)** Summary of read alignments from targeted DNA sequencing of PDXs 201.1A-Cx, 27.1A-Cx, 27.2A-Cx and 382M. PDX 201.1A-Cx does not have a structural rearrangement of the *AR* gene. The loss of reads at exons 5-7 for the other PDXs demonstrates structural rearrangements for these tumours. **(D)** Summary of read alignments from deep targeted DNA sequencing of PDX 382M.

**
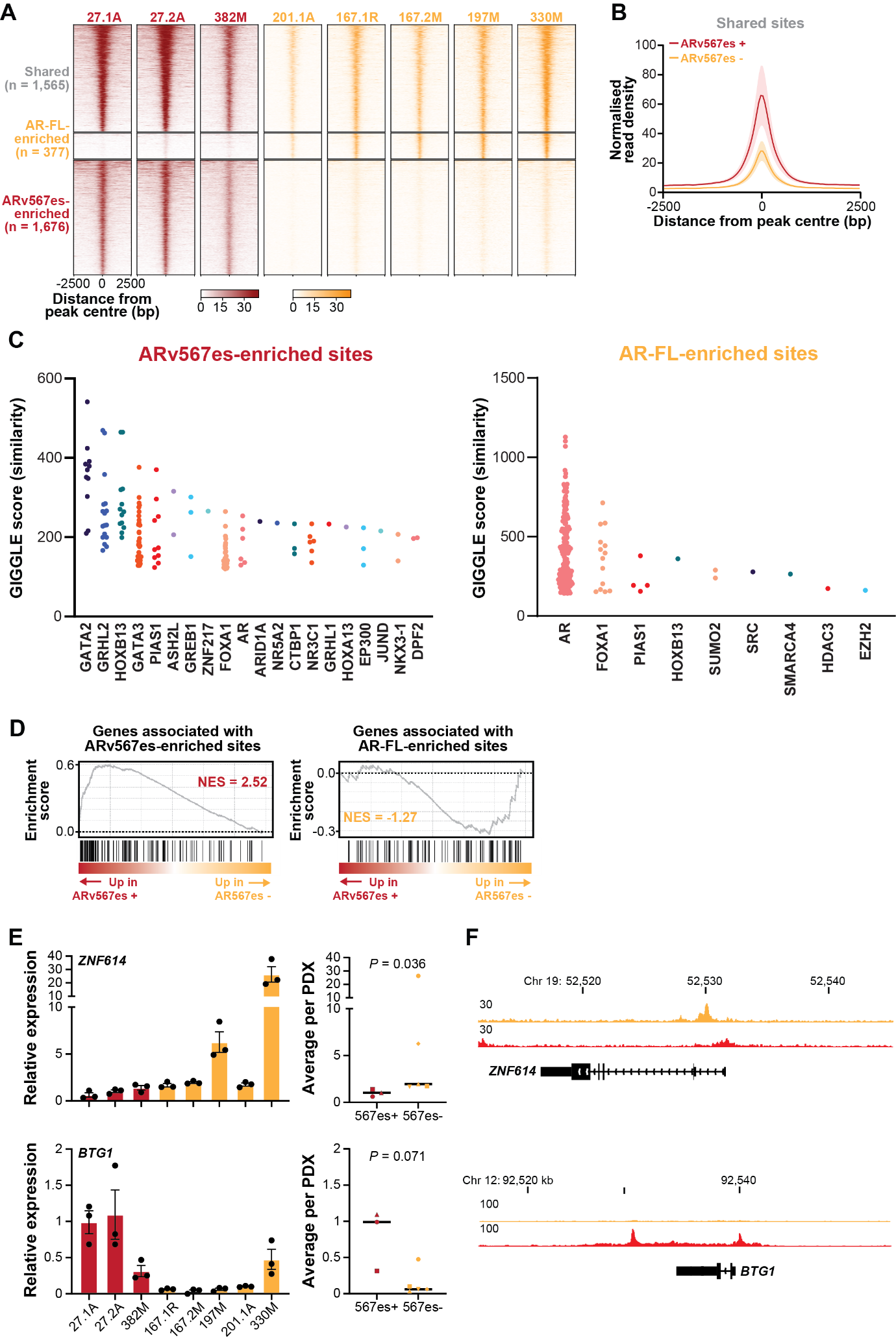
**

**Supplementary Figure 4. Comparison of ARv567es and AR-FL transcriptional activities in metastatic prostate cancer. (A)** Heat maps indicating AR ChIP-seq signal intensity in each patient-derived xenograft model at sites shared between ARv567es-negative tumours and ARv567es-positive tumours, enriched in ARv567es-positive tumours (“ARv576es-enriched”) or enriched in ARv567es-negative tumours (“AR-FL-enriched”). **(B)** Read density plot for AR ChIP-seq data proximal to sites shared between ARv567es-negative tumours and ARv567es-positive tumours. Data represent the average of the 3 ARv567es-positive and the 5 ARv567es-negative models. **(C)** GIGGLE plot showing overlap between ARv567es-enriched sites (left) and AR-FL-enriched sites (right) and publicly available cistrome data for transcription factors and epigenetic regulators. Each point indicates an individual cistromic dataset. **(D)** Gene set enrichment analysis showing that genes associated with ARv567es-enriched sites are upregulated in ARv567es-positive tumours, and vice versa. (**E**) Validation of candidate differentially expressed genes between ARv567es-positive and ARv567es-negative PDXs. Graphs show the quantitative RT-PCR data for the relative expression for each PDX (left panels; n=3 generations per PDX) and the average relative expression per PDX (right panels; unpaired Mann-Whitney test). All data are normalised to average expression in PDX 27.2A. (**F**) AR ChIP-seq data showing the average peaks for ARv567es-positive (red) and ARv567es-negative (yellow) PDXs at candidate genes.

**
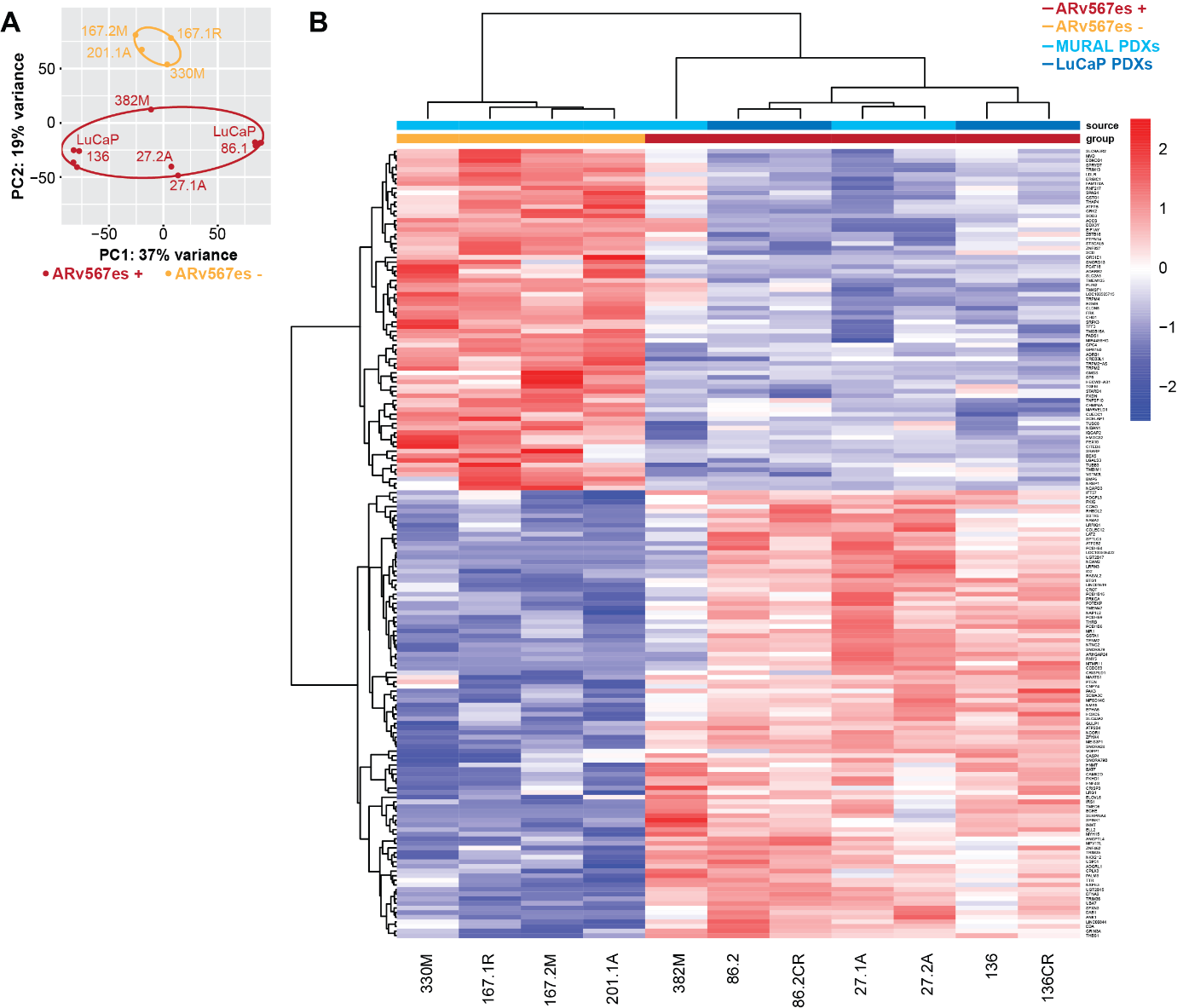
**

**Supplementary Figure 5. Validation with independent ARv567es models. (A)** PCA plot of RNA-seq data from MURAL and LuCaP PDXs (n=4 samples/PDX for LuCaP PDXs). (**B**) Heatmap of differentially expressed genes between ARv567es-positive and ARv567es-negative PDXs across the MURAL and LuCaP collections


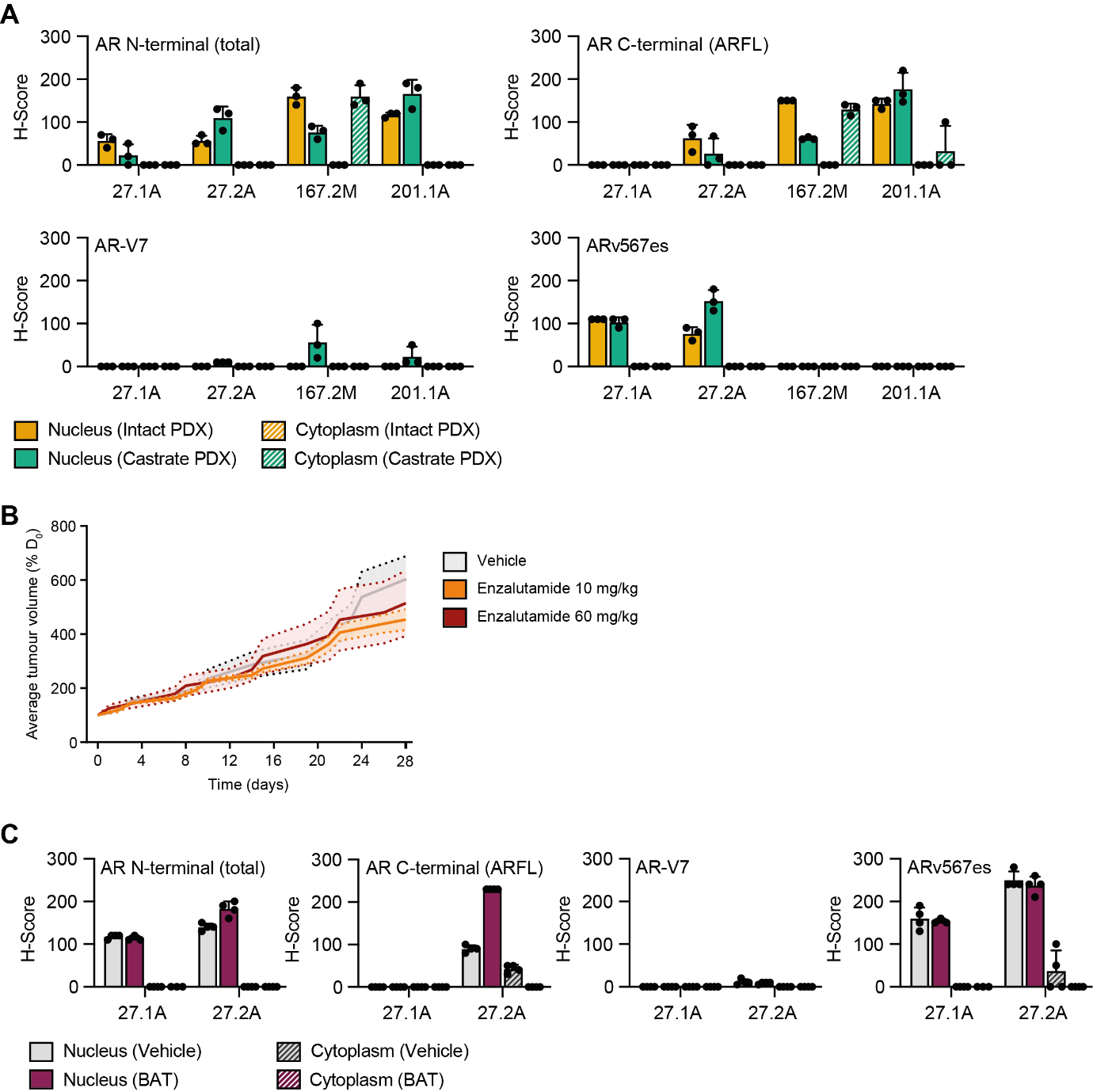


**Supplementary Figure 6. Quantification of AR staining and response to enzalutamide treatment.** (A) Separate H scores for nuclear and cytoplasmic staining with antibodies directed to the AR N-terminal, C-terminal, AR-V7 and ARv567es. The graphs represent PDXs from intact mice with testosterone implants versus castrated mice. (B) Average tumour volume relative to day 0 (%D_0_) for PDX-27.2A treated with vehicle control (grey), 10 mg/kg enzalutamide (orange) or 60 mg/kg enzalutamide (red). The solid line shows the average volume, while shaded areas represent the SEM (n=3-4 mice per group). (C) Separate H scores for nuclear and cytoplasmic staining for PDX-27.1A and 27.2A treated with vehicle or BAT for 24 hrs (n=3-4 grafts per treatment group). For panel A and C, the H scores for nuclear and cytoplasmic staining were added together to determine the total H scores depicted in Figure 3D and 4D.
